# Case-control study on factors associated with a decreased milk yield and a depressed health status of dairy herds in northern Germany

**DOI:** 10.1186/s12917-019-2190-4

**Published:** 2019-12-05

**Authors:** Katharina Charlotte Jensen, Cornelia Frömke, Bettina Schneider, Phuong Do Duc, Frieder Gundling, Katrin Birnstiel, Franziska Schönherr, Theresa Scheu, Anika Kaiser-Wichern, Svenja Woudstra, Christian Seyboldt, Martina Hoedemaker, Amely Campe

**Affiliations:** 1 Department of Biometry, Epidemiology and Information Processing, WHO Collaborating Center for Research and Training for Health at the Human-Animal-Environment Interface, and Clinic for Cattle, University of Veterinary Medicine Hannover, Foundation, Hannover, Germany; 2 Clinic for Cattle, University of Veterinary Medicine, Foundation, Hannover, Germany; 3 Present address: Faculty III, Department Information and Communication, University of Applied Sciences and Arts Hannover, Hannover, Germany; 4 Present address: Educational and Research Centre for Animal Husbandry, Hofgut Neumuehle, Muenchweiler a.d. Alsenz, Germany; 5 Present address: Lely Deutschland GmbH, Waldstetten, Germany; 6 Institute of Food Quality and Food Safety, University of Veterinary Medicine Hannover, Hannover, Germany; 7 Friedrich-Loeffler-Institut, Institute of Bacterial Infections and Zoonoses, Jena, Germany

**Keywords:** Dairy cow, Health management, Housing, Hygiene, Feeding management

## Abstract

**Background:**

In the past years, it became apparent that health status and performance differ considerably within dairy farms in Northern Germany. In order to obtain clues with respect to possible causes of these differences, a case-control study was performed. Case farms, which showed signs of health and performance problems, and control farms, which had none of these signs, were compared. Risk factors from different areas such as health management, housing, hygiene and nutrition were investigated as these are known to be highly influential. The aim of this study was to identify major factors within these areas that have the strongest association with health and performance problems of dairy herds in Northern Germany.

**Results:**

In the final model, a lower energy density in the roughage fraction of the diet, more pens with dirty lying areas and a low ratio of cows per watering spaces were associated with a higher risk for herd health problems. Moreover, case farms were affected by infections with intestinal parasites, lungworms, liver flukes and Johne’s Disease numerically more often than control farms. Case farms more often had pens with raised cubicles compared to the deep bedded stalls or straw yards found in control farms. In general, the hygiene of the floors and beddings was worse in case farms. Concerning nutrition, the microbiological and sensory quality of the provided silages was often insufficient, even in control farms. Less roughage was provided to early lactating cows and the feed was pushed to the feeding fence less frequently in case farms than in control farms.

**Conclusions:**

The results show that milk yield and health status were associated with various factors from different areas stressing the importance of all aspects of management for good animal health and performance. Moreover, this study confirmed well-known risk factors for health problems and performance losses. These should better be taken heed of in herd health management.

## Background

With an amount of approximately 32,600,000 t milk produced in 2016, Germany produced more milk than any other country in the European Union. For Germany, dairy industry is the most important sector of agricultural industry. Dairy farming experienced a substantial structural change in Germany in the last decades. From 2010 to 2016, the number of farms dropped by approximately 23%, while the number of cows stayed nearly the same [1]. This rapid change confronted the farmers with new tasks, such as human resources management. The mean milk yield per cow and year rose from 6208 kg in 2000 to 8059 kg in 2016 [2]. The higher milk yield challenges the farmers with higher demands concerning housing and feeding.

Since the 1990ies, it was reported that health and performance problems above-average occurred in a substantial number of dairy farms in (Northern) Germany [3, 4]. First, some farmers and veterinarians assumed infectious agents to cause these problems. In particular, *Clostridium botulinum* (*C. botulinum*) was supposed to be the main cause of these problems and a new form of toxicoinfection named chronic or visceral botulism was postulated [3]. This issue has initiated a very controversial debate among the veterinary and dairy community in Germany [5]. For this reason, an extensive case-control study was conducted to detect an association between poor health status and *C. botulinum* and its toxin, respectively. However, no association could be substantiated [6, 7]. Under the light of the undoubtable existence of severe health problems in dairy herds, the question of possible other causes was still unanswered. As no pathognomonic clinical picture could be observed, but many different symptoms [3], various causes had to be considered. Therefore, a systematic examination of herd health management was necessary.

For this reason, within the case-control study on *C. botulinum*, also different areas of dairy management were analyzed to identify possible causes for problems with health and milk yield in an explorative approach. Well known risk factors from the areas health management, housing, hygiene and nutrition were considered as they may have a substantial effect on milk yield and health status of dairy herds. These risk factors interact in a complex system and can influence several outcomes. To explore the current situation of this complex system, it was examined how risk factors from different areas of farm management were associated with a general, composed endpoint indicating health and performance problems. The hypothesis was to identify associations between risk factors from the areas health management, housing, hygiene and nutrition and a decreased milk yield, an increased mortality, an increased culling rate, an increased number of downer cows and farmers´ or veterinarians´ impression of herd health problems of dairy herds in Northern Germany.

## Results

### Participants

In the statistical analyses, 92 farms were included, of which 45 farms were case farms and 47 farms were control farms. Case farms were defined to fulfil at least 3 of the following five criteria: reduced milk yield (> 15% for at least three months compared to the milk yield of the year before), increased mortality (> 5% of the herd during the last year), increased culling rate (> 35% of the herd during the last year or an increase of > 10% compared to the year before), increased number of downer cows (> 10% of the herd during the last year) and farmers´ or veterinarians´ impression of herd health problems. Control farms fulfilled none of these criteria. Most farms kept mainly Holstein Friesians, but 11 farms (8 control and 3 case farms) kept Red Holsteins or crossbreeds. Milk yield was lower on case farms (case farms: 22.8 kg per cow and day, control farms: 26.0 kg per cow and day), due to the first inclusion criterion, the decreased milk yield.

### Risk factors

The results of descriptive analyses and single-factorial logistic regression analyses are shown in Tables 1 and 2. The results of the final multifactorial analysis are shown in Table 3. Factors from three of four areas of farm management (housing, hygiene and nutrition) revealed a statistically significant relationship with the current health and performance status in the investigated farms in multifactorial modeling.

**Table 1:** Descriptive and single-factorial analyses of risk factors for health and performance problems in dairy farms in Northern Germany (qualitative variables); a varying number of farms is due to missing values

Risk factors	Category	Cases		Controls		Single factorial analyses			
		N	%	N	%	OR	LCL	UCL	*P*
Health Management									
Infectious diseases									
Positive for liver flukes	No^1^	31	68.9	38	80.9	1			
	Yes	14	31.1	9	19.2	1.91	0.73	4.99	0.1887
Positive for lungworms	No^1^	28	62.2	37	78.7	1			
	Yes	17	37.8	10	21.3	2.25	0.89	5.65	0.0855
Positive for intestinal parasites	No^1^	21	46.7	32	68.1	1			
	Yes	24	53.3	15	31.9	2.44	1.04	5.69	0.0394
Positive for MAP	No^1^	40	88.9	45	95.7	1			
	Yes	4	8.9	1	2.1	4.49	0.48	41.79	0.1873
Claw Health									
Claw with high-grade dermatitis digitalis	No^1^	22	48.9	26	55.3	1			
	Yes	23	51.1	21	44.7	1.29	0.57	2.94	0.5374
Number out of ten examined cows with poor claw condition	No cow^1^	27	60.0	29	61.7	1			0.1426^2^
	One cow	7	15.6	13	27.7	0.58	0.20	1.67	0.3104
	> one cow	11	24.4	5	10.6	2.36	0.73	7.69	0.1533
Frequency of herd claw trimming	monthly or quarterly^1^	3	6.7	5	10.6	1			0.7800^2^
	Half-yearly	23	51.1	22	46.8	1.74	0.37	8.18	0.4815
	> half-yearly/ irregularly	19	42.2	20	42.6	1.58	0.33	7.56	0.5645
Housing									
Stocking density									
Average ratio of cows per watering place	≤ 1 ^1^	15	33.3	9	19.2	1			0.1033^2^
	1.01–1.5	16	35.6	27	57.5	0.36	0.13	1.00	0.0498
	> 1.5	14	31.1	11	23.4	0.77	0.24	2.40	0.6438
Average ratio of cows per feeding place	≤ 1 ^1^	14	31.1	10	21.3	1			0.3551^2^
	1.01–1.5	25	55.6	26	55.3	0.69	0.26	1.83	0.4523
	> 1.5	6	13.3	11	23.4	0.39	0.11	1.41	0.1501
Average ratio of cows per cubicle	≤ 1 ^1^	21	46.7	21	44.7	1			
	> 1	24	53.3	26	55.3	0.92	0.41	2.10	0.8484
Comfort of cubicles									
% of pens with raised cubicles	No pen^1^	9	20.0	15	31.9	1			0.0465^2^
	1–99% of pens	12	26.7	19	40.4	1.06	0.35	3.16	0.9270
	All pens	24	53.3	13	27.7	3.08	1.06	8.94	0.0390
Number of pens with no bedding material in cubicles	No pen^1^	29	64.4	32	68.1	1			
	≥ 1 pen	16	35.6	15	31.9	1.18	0.50	2.80	0.7120
Number of pens with no bedding material nor rubber mats in cubicles	No pen^1^	40	88.9	38	80.9	1			
	≥ 1 pen	5	11.1	9	19.2	0.53	0.16	1.72	0.2885
Dimensions of cubicles									
Average height of neck rail of cubicles > 115 cm	Yes^1^	26	57.8	26	55.3	1			
	No	19	42.2	21	44.7	0.91	0.40	2.07	0.8121
Average width of cubicles > 120 cm	Yes^1^	0	0.0	0	0.0	no logistic regression possible			
	No	47	100.0	47	100.0				
Average distance from neck rail to curb > 195 cm	Yes^1^	33	73.3	36	76.6	1			
	No	12	26.7	11	23.4	1.19	0.46	3.06	0.7181
*Floors*									
% of pens with slippery floors	None^1^	21	46.7	20	42.6	1			0.4259^2^
	1–49% of pens	5	11.1	10	21.3	0.48	0.14	1.64	0.2394
	≥ 50% of pens	19	42.2	17	36.2	1.06	0.43	2.61	0.8914
Number of pens with damaged floors	No pen^1^	36	80.0	42	89.4	1			
	≥ 1 pen	9	20.0	5	10.6	2.10	0.65	6.84	0.2180
Hygiene									
% of pens with dirty or very dirty floors	0–49% of pens^1^	5	11.1	14	29.8	1			0.0481^2^
	50–99% of pens	15	33.3	17	36.2	2.47	0.72	8.49	0.1511
	All pens	24	53.3	16	34.0	4.38	1.32	14.50	0.0158
% of pens with dirty or very dirty lying areas	None^1^	12	26.7	25	53.2	1			0.0070^2^
	1–49%	8	17.8	11	23.4	1.52	0.48	4.75	0.4756
	≥ 50%	24	53.3	11	23.4	4.73	1.76	12.72	0.0020
Nutrition									
*Feeding management*									
Frequency of daily feed delivery felc^3^	≥ twice a day^1^	17	37.8	20	42.6	1			
	< twice a day	28	62.2	27	57.5	1.22	0.53	2.81	0.6407
Frequency of pushing the feed back to the fence felc^3^	≥ 5 times a day^1^	3	6.7	8	17.0	1			0.1318^2^
	4 times a day	12	26.7	15	31.9	2.40	0.53	10.88	0.2562
	3 times a day	14	31.1	15	31.9	2.80	0.63	12.50	0.1773
	< 3 times a day	16	35.6	8	17.0	6.00	1.26	28.50	0.0242
*Silage quality*									
High-grade mildewed silage or a silage with decomposition or loss of structure	No^1^	14	31.1	23	49.0	1			
	Yes	31	68.9	24	51.1	2.12	0.91	4.97	0.0834
Silage with abnormal dry matter content	No^1^	30	66.7	33	70.1	1			
	Yes	15	33.3	14	29.8	1.18	0.49	2.84	0.7145
Grass silage with crude ash content (> 8%)	No^1^	3	6.4	1	2.1	1			
	Yes	44	93.5	46	97.9	1.47	0.23	9.21	0.6834
Grass silage with pH-value > 4.7 or corn silage with pH-value > 4.2	No^1^	43	95.6	45	95.7	1			
	Yes	2	4.4	2	4.3	1.05	0.14	7.76	0.9645
Silage with microbiological deviations	No^1^	9	20.2	12	25.5	1			
	Yes	36	80.0	34	72.3	1.37	0.51	3.66	0.5282
Crude fiber									
% of the herd with milk fat < 3% in the last DHI data	< 3%	30	66.7	28	59.6	1			0.6034^2^
	3–5%	9	20.0	9	19.2	0.93	0.32	2.69	0.8983
	> 5%	6	13.3	10	21.3	0.56	0.18	1.74	0.3170
Crude fiber per kg DM in the diets < 18% (PMR) or < 16% (TMR)	No^1^	18	40.0	16	34.0	1			
	Yes	27	60.0	31	66.0	0.77	0.33	1.81	0.5547
Confounders									
Season of the farm visit	Nov-Apr^1^	15	33.3	26	55.3	1			
	May-Oct	30	66.7	21	44.7	2.48	1.06	5.77	0.0356
Access to pasture	No^1^	9	20.0	16	34.0	1			
	Yes	36	80.0	31	66.0	2.06	0.80	5.32	0.1337

**Table 2:** Descriptive and single-factorial analyses of risk factors for health and performance problems in dairy farms in Northern Germany (quantitative variables; no missing values in either status group)

Variable	N	Cases			N	Controls			Single factorial analyses			
		Mean	Median	CV		Mean	Median	CV	OR	LCL	UCL	*P*
Crude fiber												
% of the herd with fat-protein-quotient < 1 in the DHI data	45	7.8	6.7	81.5	47	8.7	7.3	70.2	0.98	0.91	1.05	0.5228
Ratio of roughage in the complete diets felc^1^ based on DM content	45	58.6	58.6	15.8	47	58.2	58.0	12.4	1.01	0.97	1.05	0.5035
Energy density												
Energy density in the roughage diets in MJ NEL/kg DM felc^1^	45	6.3	6.3	3.2	47	6.4	6.4	3.5	0.06	0.01	0.51	0.0091
Energy density in the complete diets in MJ NEL/kg DM felc^1^	45	6.9	6.9	3.3	47	7.0	7.0	2.7	0.12	0.01	1.02	0.0519
Quantity of feed												
Roughage per cow and day in kg DM felc^1^	45	13.4	13.2	17.4	47	14.3	14.2	12.7	0.81	0.65	1	0.0541
Confounder												
Herd size (lactating and dry cows)	45	118.9	108.0	58.9	47	144.8	120.0	47.0	0.99	0.99	1.00	0.0867

^1^*felc* for early lactating cows (first 100 days in milk)

*OR* Odds Ratio

*LCL* Lower Confidence Level

*UCL* Upper Confidence Level

*DHI* Dairy Herd Improvement

**Table 3:** Results of multifactorial analyses: significant risk factors for health and performance problems in dairy farms in Northern Germany

Risk factors	Category	OR	LCL	UCL	*P*
Average ratio of cows per watering place	≤ 1 ^1^	1			0.0303^2^
	1.01–1.5	0.208	0.06	0.69	0.0122
	> 1.5	0.549	0.15	1.95	0.7308
% of pens with dirty or very dirty lying areas	None^1^	1			0.0114^2^
	1–49%	1.58	0.45	5.54	0.5431
	≥ 50%	5.08	1.72	15.01	0.0062
Energy density in the roughage diets in MJ NEL/kg DM felc^3^	quantitative	0.045	< 0.01	0.46	0.0088

^1^Reference category

^2^global *p*-value

^3^*felc* for early lactating cows (first 100 days in milk)

*OR* Odds Ratio

*LCL* Lower Confidence Level

*UCL* Upper Confidence Level

#### Health management

Herds of case farms were numerically more often infected with liver flukes, lungworms, *Mycobacterium avium ssp. paratuberculosis* (MAP) and intestinal parasites than herds of control farms (Table 1). In the multifactorial model, these risk factors were not statistically significant.

Lameness was a serious problem in case farms [8]. Nevertheless, no relevant differences between case- and control farms were detected concerning the claw trimming interval, claw condition, and presence of dermatitis digitalis.

#### Housing

Regardless of the status group, more than 50% of farms had more cows than cubicles in pens. Pronounced overcrowding concerning the feeding spaces (> 1.5) occurred numerically more often in control than in case farms. Case farms had less often a ratio of 1 to 1.5 and more often had a good (< 1) or worse (> 1.5) ratio. This finding was significant in the multifactorial model.

Regardless of the health and performance status, only few farms used neither bedding material, mats nor mattresses. However, the more pens with raised cubicles (cubicle without deep bedding with or without mat or mattress) were apparent on a farm the higher was the probability of health and performance problems (Table 1). This finding was only significant in single-factorial analysis. Regarding the dimensions of the cubicles, no statistically significant or relevant differences between the status groups could be revealed.

#### Hygiene

Both locations for which the hygienic conditions were evaluated (lying areas and floors) showed statistically significant associations with the herd health status in single-factorial analyses. In multifactorial modeling, the probability of health and performance problems increased by 5.1-times when more than 50% of the lying areas were soiled with manure (Table 3).

#### Nutrition

The more frequently feed was pushed back to the fence for early lactating cows the less probable the farm experienced health and performance problems, yielding in a 6-fold increase of the probability to have health and performance problems when feed was pushed back to the fence less than 3-times per day (single-factorial analysis; Table 1).

Silage quality regarding microbiological and sensory deviations was surprisingly deficient, even in most control farms (Table 1). The low sensory and microbial quality resulted in a lower energy density in the roughage fraction of the diet for fresh lactating cows. In the multifactorial model, a higher energy density in the roughage diets significantly decreased the probability of health and performance problems by 1.3-times per 0.1 net energy content for lactation per kilogram of dry matter (MJ NEL/kg DM) for early lactating cows (Table 3). Also the energy in the complete diet for fresh lactating cows was higher in control farms.

With regard to the crude fiber content in the diet, no significant differences were found between case and control farms.

## Discussion

### Study design

A case-control design was considered most appropriate, particularly because several risk factors could be evaluated simultaneously and in a short period of time [9, 10]. By design, case-control data are not able to prove causality. However, all factors included in the analyses were selected as their impact on the health of dairy cows was already described elsewhere.

The area under the receiver operating characteristic (ROC) curve in the multifactorial model was 0.774. Therefore, the model was sufficiently able to correctly predict the response of individual subjects [11]. Hence, a relevant effect of residual confounding was not expected.

### Risk factors

#### Health management

Case farms were numerically more often infected by parasites or MAP which might have contributed to the decreased milk yield and the increased mortality [12, 13]. Especially the control of parasites seems to have been neglected on case farms, as more than 50% of case farms had at least one fecal sample tested positive for intestinal parasites. It is hardly possible to compare these results to other studies because of the study design and the aggregation of data on farm level. However, gastrointestinal parasitism is a widespread problem in other countries, too [14].

The reasons why no differences could be detected concerning the claw health can only be assumed. However, an effect of reverse causation [9] should be taken into account, meaning that some farmers may have already addressed their lameness problems by changing the management, i.e. increasing the frequency of claw trimming to treat lameness. Reverse causation is a well-known phenomenon in case-control studies. The cause of the disease may have been long before the time, when the disease sat on and was evaluated. In the current study, possible causes or promoting factors and the herd health status were evaluated at the same time. If any changes concerning the risk factors had been made in the meantime, the true exposure status might have been obscured.

#### Housing

Overstocking was found to be a problem despite of the status group. This finding is in accordance with the study by Cook et al. [15] performed in Wisconsin. In contrast, King et al. [16] found on average less cows than cubicles per pen in farms in Canada. However, stocking rates of approximately 1.1 or higher are known to lead to decreased lying and rumination time and increased idle standing [17, 18].

In the multifactorial model, a medium stocking density concerning the watering spaces appeared to decrease the probability of chronic herd health problems in case farms compared to control farms. This finding may be due to coincidence or study design as case farms had, by definition, a higher mortality rate and higher culling rate. Therefore, by the time of investigation the stocking rates might have been lower than at the onset of health and performance problems.

The fact, that more case farms had pens with raised cubicles is in accordance with the fact that cows in case farms more often had hock lesions [8]. Hock lesions are known to be found more often in housing systems with raised cubicles [18]. Overcrowding and raised cubicles may have a negative impact on lying time [19, 20]. Impaired lying time is known to increase the risk of lameness [18, 21, 22] and may cause stress [23]. Thereby, health and performance problems could have been promoted.

Current recommendations concerning the width of the cubicles were not met by either case or control farms. This finding is in accordance with other studies performed in Europe [24, 25].

#### Hygiene

The fact that case farms had statistically significant more often soiled lying areas is in accordance with the worse hygienic conditions of affected herds compared to control farms [8]. It is common knowledge that insufficient hygiene can increase the incidence of mastitis and lameness [26, 27], which can result in higher culling rates and higher mortality. Hence, it can be suggested that the worse hygienic status may have contributed to the health and performance problems.

#### Nutrition

Results of this study emphasize the impact of feeding management (frequency of feed push-ups and feed delivery) even though the differences were not statistically significant in multifactorial modeling. Compared to the study of King et al. [16], the mean of feed push-ups was relatively low on the case as well as on the control farms.

No statistically significant differences could be detected between status groups concerning the quality of silages. However, especially the microbial status of silages and the prevalence of molds and decomposition shows room for improvement.

Concerning the feeding management it can be supposed that the lower energy density in roughage diets may have resulted in a negative energy balance for early lactating cows, which is known to promote various disorders [28, 29]. These might have contributed to the increased mortality, culling rate, rate of downer cows and farmers´ impression of a diseased herd. In addition, an energy deficiency and other deficiencies might also have contributed to the decreased milk yield.

Even though no differences could be found concerning the supply of cows with crude fiber, it still might play a crucial role on an individual farm, independent from the status group. Neutral detergent fiber (NDF) and acid detergent fiber (ADF) contents of the diets were not evaluated in this study. These values are currently not available in Germany for many supplements. Future studies should take the content of NDF and ADF into account when assessing differences between crude fiber contents of diets.

### Implications for the future

The results of the study presented here show that there is considerable room for improvement in different aspects of dairy husbandry in Northern Germany. Dairy herds with impaired health and performance differed from control herds with regard to several well-known management factors. Therefore, the following recommendations can be deduced from this study:

- Silage quality, energy density in the ration and feeding management should be checked and revised.
- The stocking rates, and type of bedding should be checked, and if necessary, improvements should be made.
- Infections with parasites should be taken into more thorough account.
- Attention should be focused on the hygiene of the environment of cows, and if necessary, manure management should be improved.

As all these factors relate to different areas of dairy husbandry, we can conclude that a systematic and professional analysis of each farm, e.g. by herd health management services, is necessary to improve performance and health. Future research and discussions should also evaluate, why some farmers were unable to implement some well-known principles of good agricultural practice. Underlying socioeconomic reasons shall be taken into account, e.g. by the use of qualitative methods. Tailored and client-centered support should be provided to farmers. In addition, stable schools, seminars on work organization, professional herd health programs or HACCP-concept based programs might be useful to support farmers [30–32].

## Conclusions

In the current study, associations between well-known risk factors from various areas of farm management and health and performance problems were observed in dairy herds in the northwest of Germany and promising intervention measures were deduced.

Risk factor analyses showed that factors from nearly all areas of farm management were associated with herd health and performance status. However, parasite control, improving silage quality, cow comfort and hygiene appeared to be the most promising measures against health and performance problems. Even though these factors are known for a long time to cause health problems, it cannot be taken as a given that farmers always succeed in fulfilling best agricultural practice. As the risk factors identified relate to different areas of dairy husbandry, we conclude that in case of herd health problems, all areas should be considered systematically, e.g. by herd health management services. Therefore, herd health analyses regarding the farm as a whole are indicated. In particular, individually fitted herd health management programs might be necessary to support farmers in overcoming herd health problems.

## Methods

### Study design

A case-control study was conducted as described by Seyboldt et al. [7] and Jensen et al. [8] Cases were defined to fulfill at least three of the following five criteria: reduced milk yield (> 15% for at least three months compared to the milk yield of the year before), increased mortality (> 5% of the herd during the last year), increased culling rate (> 35% of the herd during the last year or an increase of > 10% compared to the year before), increased number of downer cows (> 10% of the herd during the last year) and farmers´ or veterinarians´ impression of herd health problems. The controls did not fulfill any of these criteria. All farms were located in the northwest of Germany (Lower Saxony, Schleswig-Holstein, and Northern part of North Rhine-Westphalia). In addition, all participating farms had a loose housing system for lactating cows, minimum herd size of 30 cows and were participating in Dairy Herd Improvement (DHI) milk tests.

Based on the sample size of 46 case and 46 control farms, an odds ratio of ≥4 was detectable (confidence 95%, power ≥ 80%, prevalence of controls 50%; calculated using NCSS Pass®).

All farms were visited once by a team of four research veterinarians who were trained with regard to the examination and data collection processes. During the farm visit, they scored the herd for body condition, hygiene, skin lesions and lameness; interviewed the farmers regarding herd health, management and diet composition; checked the housing conditions; assessed feedstuff; and examined five cows with obvious chronic conditions as well as five cows without obvious conditions. These ten cows were selected in accordance to defined eligibility criteria [7]. If the five cows in a chronically sick condition showed lameness, they were examined in a claw trimming chute. In addition, silage, blood, feces, and bulk milk samples were taken. For all of these procedures, the four observers were trained prior to and during data collection. Standard operating procedures were used (SOPs; see Additional file 1: definition of risk factors). Different sections of data were collected by observers interchangeably. Inter-observer-reliability was not evaluated and observer effect was not considered during risk factor analyses. This was due to the a-priori training, usage of SOP’s and permanent training and supervision of the whole observer group by three different senior supervisors. Furthermore, a potential observer effect would not have affected data analyses due to the interchange between data collection parts and the fact that case and control farms were investigated by the same group of study vets, who visited every farm with a different composition of team members.

### Confounders

In addition to the evaluated risk factors, the three following confounders were studied: herd size (quantitative), season during which the farm visit took place (summer: May–October; winter: November–April), and access to a pasture (yes, at least seasonally; no, not at all). Descriptive statistical analyses, as well as single and multifactorial regression analyses, were utilized to assess the association of these confounding variables with case-control status.

Although the study region was chosen to reach a homogeneous study population with similar farm structures [33] and the definition of further eligibility criteria, structural differences were found: Slightly more case than control farms were visited during summer (Table 1). Case farms had fewer cows than control farms (Table 2) and cows from case farms more often had access to pastures (Table 1). These findings indicate a more extensive management system in case farms as compared to control farms. This is consistent with DHI data from Schleswig-Holstein, where larger farms had a lower culling rate and lower mortality [34]. The confounders did not show a significant impact in the multi-factorial modelling.

### Risk factors

The study veterinarians were asked, what they think which risk factors contribute to the fulfilment of the inclusion criteria on case farms. Based on their answers, four areas with a varying number of risk factors were identified, such as health management (including the sub-areas of infectious diseases and claw health), housing (including the sub-areas of stocking density, dimensions of cubicles, comfort of cubicles, and floors), hygiene, and nutrition (including the sub-areas of feeding management, silage quality, energy density, quantity of roughage, and crude fiber). Risk factors were aggregated at the farm level. An overview of each of the variables investigated is given in the following passages. More detailed definitions of the risk factors and references are provided in the Additional file 1 (definition of risk factors).

#### Health management

For the detection of liver flukes, lungworms and intestinal worms, feces samples from the ten cows that were examined clinically were tested for eggs via flotation, separately. In addition, a bulk milk sample was checked for antibodies against liver flukes (IDEXX©). For the detection of lungworms, serum samples of the ten examined cows were tested for antibodies. For the detection of MAP, feces samples from the five cows that were in a poor condition and five cows that were in good condition were pooled separately and examined via microbial culture. A farm was considered positive when at least one result from at least one sample was positive. The laboratory analyses were performed by different commercial service providers.

With regard to claw health, the frequency of herd claw trimming (quarterly or more often, every 6 months, longer than every 6 months or irregularly) was evaluated in the analyses. In addition, the number out of the ten examined cows with poor claw condition (no cows, one cow, more than one cow) was recorded, and whether high-grade dermatitis digitalis was found on at least one claw of the examined cows that showed lameness was also included in the statistical analyses (yes or no).

#### Housing

To evaluate the stocking density, the average ratio of the numbers of cows in the pen per cubicle (≤1 = no overcrowding; > 1 = overcrowding), feeding spaces and watering places (< 1 = no overcrowding, 1.01–1.5 = moderate overcrowding; > 1.5 = severe overcrowding), were calculated across all pens with lactating or dry cows on the farm (disregarding calving pens or pens for sick cows). In the case of absent feeding fences, one feeding space was defined as 0.75 m of the feed alley [35]. To calculate the watering space, a cup drinker was assumed to be sufficient for eight cows. In the case of trough watering, a length of 8 cm was defined as one watering place [35].

To assess the comfort of cubicles, the number of pens with raised cubicles were counted (no pen, at least one pen but not all pens, all pens). It was also noted whether there was a pen without rubber mats or bedding material (no pen, at least one pen).

To evaluate the dimension of the cubicles, the width of cubicles (> 120 cm; yes or no), average height of neck rails (> 115 cm; yes or no), and average distance from neck rail to curb (> 195 cm; yes or no) were measured at four randomly chosen cubicles in every pen with lactating or dry cows [35]. Normally, the fourth and the fourth-to-last of the cubicles of the row next to the wall, the fourth-to-last cubicle of the middle row and the fourth cubicle of the row next to the feeding fence were measured. Firstly, the mean of cubicle sizes was calculated at pen level. Secondly, the mean of all pens with lactating or dry cows was calculated to aggregate the data at the farm level and was compared to recommendations mentioned above.

In addition, the percentage of pens with slippery floors was assessed (no pen, 1–50% of the pens, more than 50% of the pens) as well as whether or not at least one pen had damaged floors (no pen, at least one pen with damaged floors).

#### Hygiene

The percentage of pens with dirty or very dirty floors (< 50% of the pens, 50–99% of the pens, 100% of the pens) and dirty or very dirty lying areas (no pen, at least one pen, but not all pens, all pens) was calculated and included in the analyses.

#### Nutrition

To assess feeding management, the frequency of daily feed delivery and frequency of pushing the feed back to the fence for early lactating cows (first 100 days after parturition) were included in the analyses based on farmers´ statements (see Additional file 2).

Silage quality was investigated whether or not at least one silage fed to lactating or dry cows was considered to be below current recommendations for sensory status (decomposition, loss of structure or high-grade mildewed; yes or no) assessed by the study veterinarians, crude ash content in grass silages (> 8% of dry matter; yes or no), true protein content (grass silage < 50% true protein of crude protein content; yes or no), dry matter content (grass silage: < 30% or > 40% or corn silage: < 28% or > 35%; yes or no), pH-value (grass silage: > 4.7 or corn silage: > 4.2; yes or no), and microbiological deviations (assessment based on recommendations by VDLUFA [36]; at least one silage with profound variation; yes or no). The analyses of the silages concerning the ingredients and the microbiological status were performed by an accredited service provider.

During the interview the farmer was asked for the composition of the diet for fresh lactating cows. Diets were calculated based on farmers´ statements using Futter R® (dsp agrosoft). For the silages, the results of the laboratory analyses of the sample taken at the farm visit were used. The declaration of concentrates and supplements was assumed as stated at the product or its delivery receipt [37]. The energy density in the roughage diets (silage, hay, straw) was calculated as composite in the diet for early lactating cows. In addition, the energy density in the whole diet (with concentrates and other feedstuff) for early lactating cows was calculated. Both variables were measured as net energy content for lactation (MJ NEL) per kilogram of dry matter (DM). Additionally, the quantity of fed roughage (kilogram of DM per cow per day; quantitative) for early lactating cows was included in the analysis.

With regard to the potential lack of crude fiber, the ratio of crude fiber within the diet [< 16% for TMR (total mixed ration), < 18% for PMR (partial mixed ration; crude fiber was regarded in the fed ration without individual concentrate supply); yes or no] and ratio of roughage to the whole diet (%; quantitative) were calculated for early lactating cows. Additionally, the percentage of cows in the herd with a fat content < 3% in milk (< 3%; 3–5% or >  5% of the herd) and a fat-protein-quotient < 1 (%; quantitative) of the last DHI milk recording before the farm visit were evaluated.

### Statistical analysis

Statistical analyses were performed as described in detail by Jensen et al. [8]. After entry into a relational SQL online study database, all analyses were conducted using SAS 9.3® (SAS Institute Inc., Cary, NC, USA). Data were checked for plausibility and missing values. Variables were aggregated at the farm level (statistical unit) as described above and in the Additional file 1 (definition of risk factors). Overall, only nine data points were missing, indicating excellent data quality.

First, a descriptive analysis was performed stratified by case- and control-status. Then, the linearity of the relationship between the quantitative variables and the logit of the case control status was evaluated. Linearity was confirmed graphically using R®, version 3.1.1 (R Foundation for Statistical Computing, Vienna, Austria). Two variables (ratio of roughage to the whole ration for early lactating cows and quantity of fed roughage) had a quadratic relationship to the logit of the health status. The quadratic terms of these two variables were included in the statistical analyses. If no quadratic or linear relationship was found, the variables were categorized. Associations among risk factors were investigated using Cramer’s V (cut-off: 0.7), Spearman’s rank correlation coefficient (cut-off: |0.8|) or analyses of variance (cut-off for coefficient of determination: 0.64). No association between risk factors was beyond these cut-off values. Therefore, no risk factor was excluded from further analyses. After the tests for association among the risk factors, a single-factorial logistic regression was performed. Variables with *P* < 0.2 were included in a multifactorial logistic regression analysis. To achieve an informative model, variables in the multifactorial model were excluded using stepwise backward selection, if the corresponding *P* value was greater than 0.05. The correlation matrix of the predictors was investigated to review the associations in the final statistical models. Two-way interactions among the risk factors were included in the backward-selected model and checked for statistical significance with *P* < 0.1. After backward selection of the interactions, no interactions with *P* < 0.1 remained in the model.

ROC curves were computed for the multifactorial model assessing the performance of the model. Due to the explorative nature of this study, a multiplicity correction was omitted [38].

## Supplementary information

**Additional file 1.** Definition of risk factors; the table describes the source and the definition of the risk factors

**Additional file 2.** Questionnaire feeding management; the questionnaire was used to record the feeding management of dry and lactating cows

## Abbreviations

ADF: Acid detergent fiber
*C. botulinum*: *Clostridium botulinum*
DHI: Dairy Herd Improvement
felc: For early lactating cows (first 100 days in milk)
LCL: Lower Confidence Level
MAP: *M. avium* ssp. *Paratuberculosis*
MJ NEL/ kg DM: Net energy content for lactation per kilogram of dry matter
NDF: Neutral detergent fiber
OR: Odds Ratio
PMR: Partial mixed ration
ROC: Receiver operating characteristic
SOP: Standard Operating Procedure
TMR: Total mixed ration
UCL: Upper Confidence Level
