# Supplementary material for: Case-control study on factors associated with a decreased milk yield and a depressed health status of dairy herds in northern Germany
Source: BMC Vet Res. 2019 Dec 5;15:442. doi: 10.1186/s12917-019-2190-4 (PMC6896782; doi:10.1186/s12917-019-2190-4)
Supplement: Supplementary file 1 — Additional file 1. Definition of risk factors; the table describes the source and the definition of the risk factors [file 12917_2019_2190_MOESM2_ESM.pdf]

Project: Visceral botulism in dairy cows

Date: **Farm ID:**

Study Vet: **Group: dry cows**

DHI-Number:

A1: Are there any groups of cows in the same stage of lactation, which are fed differently? For instance, are there fresh milking cows in different pens, which are fed differently or are there dry cows on pasture and in the stable?

No ◯

Yes ◯
→ *if this is the case, fill this questionnaire twice and describe the situation briefly:*

<u>B: Feeding of dry cows</u>

B1: Dry cows are kept in the following pens (names as in the sketch):

B2: Is there a 2-phase nutritional strategy for dry cows?

Yes ◯

No ◯ → then early dry group (EDG) = close up group (CUG)

don´t know ◯

B3: Do you (or somebody else) calculate the rations for dry cows? If yes, who does it?

	**EDG**	**CUG**
Yes, done by the farmer	◯	◯
Yes, done by an advisor	◯	◯
Yes, done by a vet	◯	◯
Yes, done by the chamber of agriculture	◯	◯
Yes, done by someone else	◯	◯
No	◯	◯
don´t know	◯	◯

B4: How do you determine the requirements of the dry cows?

	EDG	CUG
Based on tables	◯	◯
Estimation	◯	◯
don´t know	◯	◯

B5: How do you measure the amount of food for dry cows?

	EDG	CUG
weighed	◯	◯
estimated	◯	◯
don´t know	◯	◯

B6: What kind of ration is fed to the dry cows?

	EDG	CUG
Total mixed ration	◯	◯
Mixed ration plus individual amount of concentrates	◯	◯
No mixed ration	◯	◯
don´t know	◯	◯

B7: Do the dry cows have access to food all the time?

	EDG	CUG
Yes	◯	◯
No	◯	◯
don´t know	◯	◯

B8: How often do you feed the dry cows? **EDG** **CUG**

_____ _____ times per day

B9: How often do you push the food back to the fence? **EDG** **CUG**

_____ _____ times per day

B10: How much of the food remains in the feeding trough? **EDG** **CUG**

_____ _____ %

B11: Do you feed toxin binders to your dry cows?

	EDG	CUG
Yes	◯	◯
No	◯	◯
don´t know	◯	◯

B12: What other feeding components than silage are fed to the dry cows?

	EDG	CUG
Pasture	◯	◯
Corn Cob Mix	◯	◯
Straw	◯	◯
Grains	◯	◯
Hay		
Grass	◯	◯
Alfalfa	◯	◯
Concentrates		
manually	◯	◯
via transponder	◯	◯
in the milking parlour	◯	◯
Protein supplements		
rapeseed	◯	◯
soy	◯	◯
misc.	◯	◯
minerals	◯	◯
pressed pulp	◯	◯
spent/ brewers´ grains	◯	◯
potato pulp	◯	◯
potatoes	◯	◯
beets/ turnips	◯	◯
Misc.:______________________________	◯	◯

B13: Do dry cows have access to water, which is not of drinking water quality?

	EDG	CUG
Yes, always	◯	◯
Yes, on a regular basis	◯	◯
Yes, seasonally	◯	◯
Yes, rarely	◯	◯
No	◯	◯
don´t know	◯	◯

Date: **Farm ID:**

Study Vet: **Group: lactating cows**

DHI-Number:

C: Feeding of lactating cows

C1: Milking cows are kept in the following pens (names as in the sketch):

Cows in early lactation (ELC):

Cows in mid-lactation (MLC):

Cows in late lactation (LLC):

C2: How many different feeding groups are there with lactating cows?

1-group strategy	◯
2-group strategy	◯
3-group strategy	◯

C3: Do you (or somebody else) calculate the diets for lactating cows? If yes, who does it?

	**ELC**	**MLC**	**LLC**
Yes, done by the farmer	◯	◯	◯
Yes, done by an advisor	◯	◯	◯
Yes, done by a vet	◯	◯	◯
Yes, done by the chamber of agriculture	◯	◯	◯
Yes, done by someone else	◯	◯	◯
No	◯	◯	◯
don´t know	◯	◯	◯

**C4: How do you determine the requirements of the lactating cows?**	**ELC**	**MLC**	**LLC**
Based on tables	◯	◯	◯
Estimation	◯	◯	◯
don´t know	◯	◯	◯

**C5: How do you measure the amount of food for lactating cows?**	**ELC**	**MLC**	**LLC**
weighed	◯	◯	◯
estimated	◯	◯	◯
don´t know	◯	◯	◯

**C6: What kind of ration is fed to the lactating cows?**	**ELC**	**MLC**	**LLC**
Total mixed ration	◯	◯	◯
Mixed ration plus individual amount of concentrates	◯	◯	◯
No mixed ration	◯	◯	◯
don´t know	◯	◯	◯

**C7: Do the lactating cows have access to food all the time?**	**ELC**	**MLC**	**LLC**
Yes	◯	◯	◯
No	◯	◯	◯
don´t know	◯	◯	◯

**C8: How often do you feed the lactating cows?**	**ELC**	**MLC**	**LLC**
	_____	_____	_____ times per day

**C9: How often do you push the food back to the fence?**	**ELC**	**MLC**	**LLC**
	_____	_____	_____ times per day

**C10: How much of the food remains in the feeding trough?**	**ELC**	**MLC**	**LLC**
	_____	_____	_____ %

**C11: Do you feed toxin binders to your lactating cows?**	**ELC**	**MLC**	**LLC**
Yes	◯	◯	◯
No	◯	◯	◯
don´t know	◯	◯	◯

C12: What other feeding components than silage are fed to the dry cows?

	ELC	MLC	LLC
Pasture	○	○	○
Corn Cob Mix	○	○	○
Straw	○	○	○
Grains	○	○	○
Hay			
Grass	○	○	○
Alfalfa	○	○	○
Concentrates			
manually	○	○	○
via transponder	○	○	○
in the milking parlour	○	○	○
Protein supplements			
rapeseed	○	○	○
soy	○	○	○
misc.	○	○	○
minerals	○	○	○
pressed pulp	○	○	○
spent/ brewers´ grains	○	○	○
potato pulp	○	○	○
potatoes	○	○	○
beets/ turnips	○	○	○
Misc.:______________________________	○	○	○

C13: Do lactating cows have access to water that is not of drinking water quality?

	ELC	MLC	LLC
Yes, always	○	○	○
Yes, on a regular basis	○	○	○
Yes, seasonally	○	○	○
Yes, rarely	○	○	○
No	○	○	○
don´t know	○	○	○
