# Supplementary material for: Case-control study on factors associated with a decreased milk yield and a depressed health status of dairy herds in northern Germany
Source: BMC Vet Res. 2019 Dec 5;15:442. doi: 10.1186/s12917-019-2190-4 (PMC6896782; doi:10.1186/s12917-019-2190-4)
Supplement: Supplementary file 2 — Additional file 2. Questionnaire feeding management; the questionnaire was used to record the feeding management of dry and lactating cows [file 12917_2019_2190_MOESM1_ESM.docx]

# Supplementary file 1

Definitions of the risk factors.

| Risk factor | Source of information, definition, and aggregation at the farm level | References |
| --- | --- | --- |
| **Health management** | | |
| *Infectious diseases* | | |
| Liver flukes | - Feces samples of ten cows were checked for worm eggs via sedimentation - Additionally, a bulk milk sample was tested for antibodies - A farm was defined as positive if at least one test result was positive | [1,2] |
| Lungworms | - Feces samples of ten cows were checked for worm eggs via flotation, sedimentation, and the Baermann funnel method - Additionally, serum samples of the same ten cows were checked for antibodies using ELISA - A farm was defined as positive if at least one test result was positive | [2] |
| Intestinal parasites | - Feces samples of ten cows were checked for worm eggs via flotation, sedimentation, and the Baermann funnel method - A farm was defined as positive if at least one test result was positive | [2] |
| MAP | - Two pooled samples (each of 5 fecal samples) were examined via microbial culture for MAP - A farm was defined as positive if at least one test result was positive |  |
| *Claw Health* | | |
| Dermatitis digitalis | - Examination of lame suspect cows by study veterinarians in a claw trimming chute - A farm was defined positive if at least one of these cows had M2 stage of disease (radius at least 2 cm) | [3] |
| Poor claw trimming condition | - Visual examination - Number out of ten examined cows with overgrowth of horn capsules (length>12 cm) and no sign of claw trimming |  |
| Frequency of herd claw trimming | - Interview with the herd manager/ farmer - Frequency of claw trimming performed by a technician or farmer on more than 75% of the herd |  |
| **Housing** | | |
| *Stocking density* | | |
| Number of cows per watering place | - Inspection of all pens with lactating or dry cows (except calving pen or pen for sick cows) - A cup drinker was defined to provide 8 watering spaces - 8 cm of trough were defined as one watering space - Average of all inspected pens per farm | [4] |
| Number of cows per feeding place | - Inspection of all pens with lactating or dry cows (except calving pen or pen for sick cows) - A feeding space was defined as one feeding fence or 0.75 m of feeding alley - Average of all inspected pens per farm | [4] |
| Number of cows per cubicle | - Inspection of all pens with cubicles with lactating or dry cows (except calving pen or pen for sick cows) - Average of all inspected pens per farm | [4] |
| *Comfort of cubicles* | | |
| % of pens with raised cubicles | - Inspection of all pens with lactating or dry cows - Raised cubicle: lying surface higher than floor, no deep litter |  |
| % of pens with no litter in cubicles | - Inspection of all pens with cubicles for lactating or dry cows - Proportions of cubicles with no wood shavings, straw or similar bedding material - Chalk was not defined as bedding material |  |
| Pen with neither litter nor rubber mats | - Inspection of all pens with lactating or dry cows - At least one pen were the lying surface is cement |  |
| *Dimensions of cubicles* | | |
| Height of neck rails of cubicles > 115 cm | - Four randomly chosen cubicles were measured per pen - Average of all pens of lactating or dry cows with cubicles was calculated | [4] |
| Width of cubicles > 120 cm | - Four randomly chosen cubicles were measured per pen - Average of all pens of lactating or dry cows with cubicles was calculated per farm | [4] |
| Distance from neck rail to curbs of cubicles > 195 cm | - Four randomly chosen cubicles were measured per pen - Average of all pens of lactating or dry cows with cubicles was calculated |  |
| *Floors* | | |
| % of pens with slippery floors | - Inspection of all pens with lactating or dry cows - Semi-qualitative assessment as slippery or rather slippery on different locations in the pen by boot test | [5] |
| Pen with damaged floor | - Inspection of all pens with lactating or dry cows - Floor was defined as damaged, if slats were protruding or if there were edges which could be a trip hazard for cows |  |
| **Hygiene** | | |
| Hygiene of floors | - Proportion of pens with floors scored 3 or 4 - Inspection of all pens with lactating or dry cows at two different locations - 1= completely free of dirt or has very little dirt; 2= slightly dirty; 3= mostly covered with dirt; or 4= completely covered with dirt | Adapted from Schreiner and Ruegg [6] |
| Hygiene of lying areas | - Proportion of pens with lying areas scored 3 or 4 - Inspection of all pens with lactating or dry cows at two different locations - 1= completely free of dirt or has very little dirt; 2= slightly dirty; 3= mostly covered with dirt; or 4= completely covered with dirt | Adapted from Schreiner and Ruegg [6] |
| **Nutrition** | | |
| *Feeding management* | | |
| Frequency of feed delivery | - Interview with the herd manager - Frequency of feed delivery for early lactating cows per day |  |
| Frequency of pushing the feed to the fence | - Interview with the herd manager - Frequency of pushing the feed to the fence for early lactating cows per day |  |
| *Silage quality* | | |
| High-grade mildewed silage or a silage with decomposition or loss of structure | - Visual inspection of all silages fed to lactating or dry cows - A farm was defined as positive if there was at least one silage with obvious signs of molds (discoloration and abnormal smell), loss of structure or decomposition | [7] |
| Dry matter content of silages | - “Weender Analysis” of all silages fed to lactating or dry cows - A farm was defined a positive if at least one silage had a dry matter content < 30% or > 40% (grass) or < 28% or > 35% (corn) | [7] |
| Crude ash content of grass silages | - “Weender Analysis” of all silages fed to lactating or dry cows - A farm was defined as positive, if at least one silage had a crude ash content > 8% | [7] |
| pH-value of silages | - Laboratory analyses of all grass silages fed to lactating or dry cows - A farm was defined a positive if at least one silage had a pH-value > 4.7 (grass) or > 4.2 (corn) | [7] |
| Microbiological deviations of silages | - Microbial analyses of all silages fed to lactating or dry cows - A farm was defined a positive if at least one silage showed microbiological deviations (at least tenfold exceeding of the orientation value of at least one group of microorganisms) | [8] |
| *Crude fiber* | | |
| % of the herd with a fat content < 3% in milk | - Based on last Dairy Herd Improvement test results | [9] |
| Crude fiber per kg DM in the ration (g/kg) < 18% (PMR) or < 16% (TMR) | - Proportion of crude fiber per kg DM in the diet of early lactating cows - Calculations were based on farmers´ statements on ration composition - Crude fiber content was determined based on “Weender Analysis” (silages) or declaration (concentrates), respectively | [7] |
| % of the herd with a fat-protein-quotient content < 1 in milk | - Based on last Dairy Herd Improvement test results | [9] |
| Ratio of roughage in the diets | - Percentage of silages, hay and straw of the rations (based on dry matter content) for early lactating cows based on farmers´ statements on ration composition - Amount of concentrates was assumed for average milk yield for the herd for early lactating cows |  |
| *Energy density* | | |
| Energy density in the roughage diet | - Energy density in roughage composed in the ration for early lactating cows in MJ NEL/kg DM based on farmers statement on ration composition - Energy content of silages was based on laboratory analyses of silages |  |
| Energy density in the complete diet | - Ration for early lactating cows calculated as stated by farmer in MJ NEL/kg DM - Energy content of silages was based on laboratory analyses - Energy content of concentrates was based on declaration - Amount of concentrates was assumed for average milk yield for the herd for early lactating cows |  |
| *Quantity of feed* | | |
| Roughage per early lactating cow and day in kg DM | - Amount of dry matter of roughage calculated as stated by farmer |  |
| **Confounders** | | |
| Herd size | - Dry and lactating cows - Based on DHI-data |  |
| Season of farm visit | - May to October or November to April |  |
| Access to pasture | - Lactating and/ or dry cows had access to pasture at least seasonally |  |

**References**

[1] Kuerpick B, Conraths FJ, Staubach C, Froehlich A, Schnieder T, Strube C. Seroprevalence and GIS-supported risk factor analysis of Fasciola hepatica infections in dairy herds in Germany. Parasitology. 2013 Jul;140(8):1051-60.

[2] Wacker K, Roffeis M, Conraths FJ. Cow–calf herds in eastern Germany: status quo of some parasite species and a comparison of chemoprophylaxis and pasture management in the control of gastrointestinal nematodes. J. Vet. Med. B Infect. Dis. Vet. Public Health. 1999 Sep;46(7):475-83.

[3] Holzhauer M, Bartels CJ, Döpfer D, van Schaik G. Clinical course of digital dermatitis lesions in an endemically infected herd without preventive herd strategies. Vet J. 2008 Aug 1;177(2):222-30.

[4] Lower Saxony Ministry for Rural Areas, Food, Agriculture and Consumer Protection. Animal welfare guideline of Lower Saxony for the keeping of dairy cows. 2007. http://www.laves.niedersachsen.de/download/41962/Tierschutzleitlinie_fuer_die_Milchkuhhaltung.pdf Accessed 2 Jul 2016

[5] De Kruif A, Mansfeld R, Hoedemaker M. Tierärztliche Bestandsbetreuung beim Milchrind. 3rd ed. Stuttgart, Germany: Enke; 2013.

[6] Schreiner DA, Ruegg PL. Effects of tail docking on milk quality and cow cleanliness. J Dairy Sci. 2002 Oct 1;85(10):2503-11.

[7] Meyer H, Kamphues J. Supplemente zu Vorlesungen und Übungen in der Tierernährung. 10th ed. Alfeld, Germany: Verlag Schaper; 2004.

[8] Verband Deutscher Landwirtschaftlicher Untersuchungs- und Forschungsanstalten. Mikrobiologische Verfahren. In: Band III, Die chemische Untersuchung von Futtermitteln. Speyer, Germany: VDLUFA-Verlag; 1976.

[9] Rodrigue CB, Aixen NN. The effect of fine grinding of hay on ration digestibility, rate of passage, and fat content of milk. Can. J. Anim. Sci. 1960 Jun 1;40(1):23-9.
